# Targeting TLR/IL-1R Signalling in Human Diseases

**DOI:** 10.1155/2010/674363

**Published:** 2010-04-08

**Authors:** Maria Loiarro, Vito Ruggiero, Claudio Sette

**Affiliations:** ^1^Department of Public Health and Cell Biology, University of Rome “Tor Vergata”, 00133 Rome, Italy; ^2^Laboratory of Neuroembryology, Fondazione Santa Lucia, Istituto Di Ricovero e Cura a Carattere Scientifico (IRCCS), 00143 Rome, Italy; ^3^Department of Immunology (Bldg. LABIO), R&D Sigma-tau Industrie Farmaceutiche Riunite S.p.A, Via Pontina km 30.400, 00040 Pomezia (RM), Italy

## Abstract

The members of Toll-like receptor/Interleukin (IL)-1 receptor (TLR/IL-1R) superfamily play a fundamental role in the immune response. These receptors detect microbial components and trigger complex signalling pathways that result in increased expression of multiple inflammatory genes. On the other hand, an aberrant activation of TLR/IL-1R signalling can promote the onset of inflammatory and autoimmune diseases, raising the interest in the development of therapeutic strategies for the control of their function. In this review, we illustrate the structural and functional features of TLR/IL-1R proteins and discuss some recent advances in the approaches undertaken to develop anti-inflammatory therapeutic drugs. In particular, we will focus on inhibitors, such as decoy peptides and synthetic mimetics, that interfere with protein-protein interactions between signalling molecules of the TLR/IL-1R superfamily. Given their central role in innate and adaptive immune responses, it is foreseen that pharmaceutical modulation of TLR/IL-1R signalling pathways by these drugs might yield clinical benefits in the treatment of inflammatory and autoimmune diseases.

## 1. Introduction

All living organisms are constantly exposed to pathogenic microorganisms that are present in the environment. To face this continuous challenge, evolution has selected mechanisms of immune defence to eliminate or counteract these invading pathogens [1]. In mammals, the immune response relies on complex strategies of defence consisting of two components: “adaptive immunity” and “innate immunity”. Adaptive immunity is a highly sophisticated system—observed only in vertebrates—characterized by an exquisite capacity to establish efficient memory responses to specific antigens. This system is able to anticipate subsequent encounters with pathogens and represents a potent defence against microbial infection [2]. Adaptive immunity is involved in the elimination of pathogens during the late phase of infection and is elicited by B and T lymphocytes, which utilize immunoglobulins and T cell receptors, respectively, as antigen receptors to recognize “non self” molecules. These receptors are generated through DNA rearrangement and respond to a wide range of potential antigens [3]. In contrast, the innate immunity, which was first described over a century ago, is phylogenetically conserved and is present in almost all multicellular organisms [4]. Innate immunity represents the first line of protection against the invading microbial pathogens and is mediated by phagocytes, such as macrophages and dendritic cells (DCs). Although it was initially viewed as a non specific response, innate immunity is indeed able to discriminate between “self” molecules and a variety of pathogens through the function of a small array of germline-encoded pattern-recognition receptors (PRRs). These receptors can specifically recognize conserved microbial components known as pathogen-associated molecular patterns (PAMPs) [4]. The PRRs include members of nucleotide oligomerization domain proteins, containing leucine-rich repeats (NLRs), retinoic acid inducing gene (RIG)-like helicases (RLHs), and toll-like receptors (TLRs) [5]. TLRs, which are one of the largest and best studied families of PRRs, and their signal transduction pathways are the focus of this review.

## 2. Structural Features of TLRs

TLRs are evolutionary conserved from plants to vertebrates. In mammals there are 12 identified TLRs [5]. These receptors undergo homo- or hetero dimerization to detect a wide range of PAMPs including lipids, lipoproteins, proteins, glycans, and nucleic acids [6, 7]. Exhaustive reviews covering the specificity for different ligands recognized by TLRs [8, 9] as well as the structural features of these receptors have been recently published [10, 11]. Here we will focus on the domains that characterize these receptors, with a particular attention to the TIR domain.

**Figure 1:**
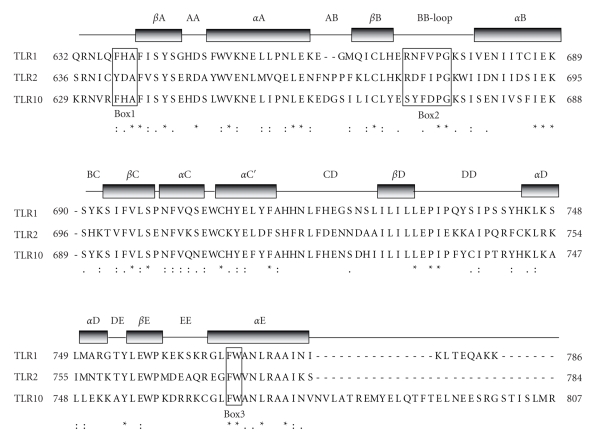
Structure of TIR domains. Sequence alignment of the TIR domains of human TLR1, TLR2, and TLR10 was performed by the ClustalW2 software. Identical residues are indicated by asterisks, while conservative or semi conservative substitutions are indicated by double-dots and single-dot, respectively. The TIR domain contains three highly conserved motifs denoted Box 1, 2, and 3 [10] that are shown in open boxes. Grey bars indicate the secondary structure of TIR domains, that are composed of five *β*-strands (from *β*A to *β*E) alternated with five *α*-helices (from *α*A to *α*E) connected by eight loops.

TLRs are characterized by two conserved regions: the extracellular leucin-rich region (LRR) and the cytoplasmic Toll/IL-1 receptor (TIR) domain. The LRR, which is deputed to recognition of the ligand, is composed of 19–25 tandem repeats of 24–29 amino acids, folded in *β*-strands and in *α*-helices that are linked by loops. The structures of TLR1, TLR2, TLR3, and TLR4 have been recently solved, leading to models that predict the mechanism of interaction with their cognate ligands [11]. The TIR domain, which shares homology with the interleukin 1 receptor (IL-1R) [12], is instead responsible for the propagation of the signal within the cell through interaction with a complex signalling cascade [8, 9, 13]. Crystallographic analyses of the TIR domain of human TLR1, TLR2 [10], and TLR10 [14] revealed that they are composed of five *β*-strands alternated with five *α*-helices connected by eight loops (Figure 1). Moreover, this domain contains three highly conserved motifs denoted Box 1, 2, and 3 [10] (Figure 1). Box 2 forms a loop connecting the second *β*-strand and *α*-helix, referred to as the BB-loop. This loop is critical for proper signalling, as single residue substitutions abolish the ability to recruit targets without changing the overall fold of the TIR domain [10]. For instance, a naturally occurring point mutation (P712H) affecting a conserved proline within the BB-loop is required for TLR4-triggered downstream signals [15]. This mutation leads to pathological consequences and renders C3H/HeJ mice hyporesponsive to lipopolysaccharide (LPS) [15]. Notably, the homologous point mutation in human TLR2 (P681H) disrupts the signal transduction induced by Gram-positive bacteria [16], confirming the critical role of the BB-loop in TLR signalling.

## 3. The TLR/IL1-R Superfamily: A Central Role for the Intracellular Adaptor Proteins

In addition to the TLR subfamily, the presence of an intracellular TIR domain is the hallmark of a large group of proteins that belong to the TLR/IL-1R superfamily [17] such as the IL-1R subfamily and the TIR-domain-containing adaptor proteins [17]. 

The IL-1R subfamily encodes nine members that are characterized by the presence of extracellular immunoglobulin-like (Ig) domains and by an intracellular TIR domain. IL-1R contains three Ig domains and, together with the highly homologous IL-1R accessory protein (IL-1RAcP), forms a receptor complex for IL-1*α* *,* IL-1*β* and IL − 1 receptor antagonist (IL-1Ra) [18]. Similarly, the IL-18 receptor (IL-18R), following binding to IL-18, forms a complex with IL-18RAcP to initiate downstream signalling. IL-1Rrp2 is the receptor for the agonists IL-1F6, IL-1F8, and IL-1F9, which also uses IL-1RAcP as a second chain [19]. Thus, IL-1RAcP appears to be promiscuous since, in addition to IL-1RI and IL-1Rrp2, it also associates with ST2, which has recently been shown to bind IL-33 [20]. IL-1R2 and SIGIRR are two inhibitory receptors, the former lacks the TIR domain whereas the latter contains a single Ig domain for the extracellular segment. The only members that still remain without any identified function in this subfamily are IL-1RAPL and TIGIRR [21].

 The third subfamily comprises several adaptor molecules: the Myeloid differentiation factor 88 (MyD88), the MyD88-adaptor-like (MAL, also known as TIRAP), the TIR-domain-containing adaptor protein inducing interferon-*β* (IFN*β*) (TRIF; also known as TICAM1), the TRIF-related adaptor molecule (TRAM; also known as TICAM2) and the sterile *α*- and armadillo-motif containing protein (SARM) [12]. These adaptors bridge the TLR/IL-1R receptors to the intracellular molecules that transduce their signals into a biological response and play a central role in innate immunity. Among them, MyD88 is without doubts the most widely utilised adaptor molecule in TLR/IL-1R signalling. It was originally identified as a novel myeloid differentiation primary response gene in M1 monoleukemic cell lines [22]. MyD88 has a modular structure consisting of an N-terminal death domain (DD) separated by a short linker region from the C-terminal TIR domain [23]. Studies on MyD88-deficient mice have clearly demonstrated that this protein is an essential component in the responses to various TLR ligands, with the sole exception of TLR3 [24–26]. The second adaptor in the subfamily to be discovered was MAL/TIRAP. MAL/TIRAP has a binding domain to phosphatidylinositol-4,5-bisphosphate (PtdIns(4,5)P2), required for its recruitment to the plasma membrane, at the N-terminus and a TIR domain at the C-terminus [27, 28]. MAL/TIRAP interacts with MyD88, and MAL-deficient mice have revealed that this adaptor is essential for MyD88-dependent signalling through TLR2 and TLR4 [29]. TRIF contains consensus TRAF6-binding motifs (T6BM) in the N-terminal region, a TIR domain and a receptor-interacting protein (RIP) homotypic interaction motif (RHIM) in the C-terminal region [30]. TRIF is the only adaptor used by TLR3, and TRIF-mutant mice display an impaired TLR3-mediated response being defective in the TLR4-mediated activation of the MyD88-independent pathway [31]. TRAM contains a TIR domain in the C-terminal region and functions exclusively in the TLR4 pathway. The N-terminus of TRAM undergoes constitutive myristoylation that mediates its association with membranes. TRAM interacts with TRIF, and TRAM-deficient mice demonstrated that this protein is also essential for the MyD88-independent pathway of TLR4 signalling [32]. Finally, SARM [33], which contains a TIR domain at C-terminus, two “sterile a” motif (SAM) protein-protein interactions domains, and an Armadillo repeat motif (ARM) [34], functions as an inhibitor of TRIF-dependent TLR signalling [35].

## 4. TLR/IL-1R Signalling Pathways

**Figure 2:**
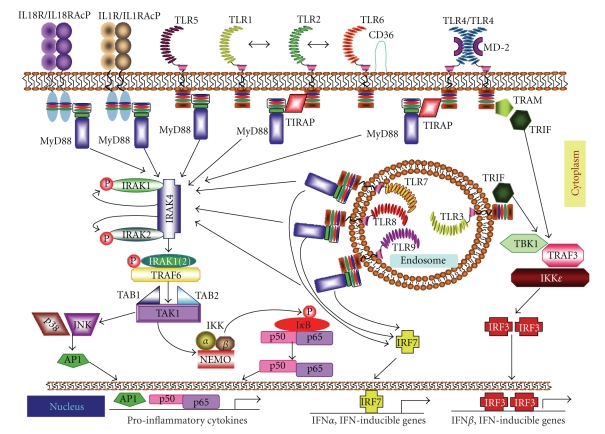
TLR/IL-1R signalling pathways. Once activated by their respective ligands, IL-1R, IL-18R, and TLRs engage with one or more adaptor proteins. These adaptors, namely, MyD88, MAL/TIRAP, TRIF, and TRAM are recruited, in various combinations, to the cytoplasmic domains of the receptors through homophilic interactions between Toll/IL-1 receptor (TIR) domains present in each receptor and each adaptor. TIRAP is required to act as a bridge for MyD88 in TLR2 and TLR4 signalling, while TRIF is used in TLR3 signalling and, in association with TRAM, in TLR4 signalling. In the MyD88-dependent pathway, MyD88 associates with IRAK4, IRAK1 and/or IRAK2. IRAK4 in turn phosphorylates IRAK1 and IRAK2 and promotes their association with TRAF6, which serves as a platform to recruit the kinase TAK1. Once activated, TAK1 activates the IKK complex, composed of IKK*α*, IKK*β*  and NEMO (IKK*γ*)*,* which catalyzes phosphorylation and subsequent degradation of I*κ*B rendering NF-*κ*B (i.e., p50/p65) free to translocate from the cytosol to the nucleus and activate NF-*κ*B-dependent genes. The transcription factor IRF7 is also activated downstream of TLRs 7, 8, and 9, leading to its translocation into the nucleus and to activation of IFN*α* and IFN-inducible genes. TLR3 and TLR4 both signal through the adaptor TRIF, which interacts with TRAF3 to activate the noncanonical IKKs, TBK1, and IKK*ε* resulting in the dimerization and activation of IRF3, which then translocates into the nucleus activating the transcription of IFN*β*  and IFN-inducible genes.

Upon recognition of their cognate ligands, TLR/IL-1R proteins homo- or hetero dimerize (TLR1/2, TLR2/6, IL-1R/IL-1RacP) and initiate a signalling cascade through recruitment of different combinations of TIR-domain-containing adaptor protein to their TIR domain, in order to turn on both common and unique pathways (Figure 2). All receptors of the superfamily, with the sole exception of TLR3, use MyD88 to initiate their signalling pathway. In some cases, MyD88 acts in concert with other adaptors, like MAL/TIRAP in the response triggered by stimulation of TLR4, TLR1/2, and TLR2/6 [12]. On the other hand, TLR3-mediated signalling requires only the adaptor molecule TRIF, which is also recruited by TLR4 in association with the other adaptor TRAM [12]. Based on the type of adaptor molecules involved, the TLR/IL-1R-induced pathways can be sub-grouped in two classes: MyD88-dependent and MyD88-independent responses.

## 5. MyD88-Dependent Signalling

**Figure 3:**
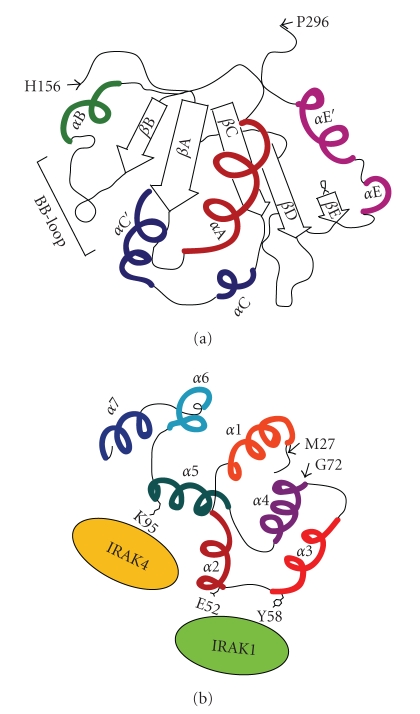
Schematic representation of the TIR and Death Domains of MyD88. (a) A schematic representation of the human MyD88 TIR domain. The TIR domain of MyD88 consists of five central *β*-strands surrounded by four *α*-helices, connected by loops [36]. It lacks the *α*-helix *α*D in the region between *β*D and *β*E strands, this region has an helical coil conformation. (b) Surface of interaction of the MyD88 Death Domain with IRAK1 and IRAK4. The region MyD88 Death Domain (DD) comprised by residues 27–72 (predicted *α*1, *α*2, *α*3 and N-terminal *α*4 helices) is required for the recruitment of IRAK1 [37]. Residues E52 and Y58 of MyD88 DD are implicated in the interaction of IRAK1 with MyD88. Moreover, residue K95 in the predicted *α*5 helix is involved in the recruitment of IRAK4 by MyD88 DD.

The MyD88 TIR domain differs from the TIR domain of TLRs, as it contains five central  *β*-strands surrounded by four *α*-helices instead of five *α*-helices [36] (Figure 3(a)). TLR/IL-1R receptors associate with MyD88 through homotypic interactions between their respective TIR domain. This interaction then allows MyD88 to recruit members of the interleukin-1 receptor-associated kinase (IRAK) family (IRAK1, IRAK2, and IRAK4) through homotypic interactions between their respective Death Domains (DDs) [38] (Figure 2). Notably, recent studies have identified critical residues in MyD88 DD required for association with either IRAK1 or IRAK4 (Figure 3(b)) [37]. Since their substitution impaired propagation of the downstream signaling response [37], it is likely that these interactions are necessary. The interaction between MyD88 and IRAK1/4 induce the formation of macromolecular complexes that ultimately impinges on TAK1 (transforming growth factor *β*-activated kinase 1) [39] and leads to activation of the transcription factor NF-*κ*B (p50/p65) [8, 9, 17, 40] (Figure 2). However, MyD88-dependent activation of NF-*κ*B can also be induced by TAK1-independent pathways, as shown by the incomplete suppression of IL-1- or LPS-induced NF-*κ*B activation in TAK1-deficient murine embryonic fibroblasts [41]. Two candidates for this TAK1-independent pathway are the mitogen-activated protein kinase kinase kinase 3 (MEKK3) and Protein kinase C (PKC) [42, 43].

TAK1 can also activate mitogen-activated protein kinases (MAPKs), such as p38 and JNK, leading to the activation of transcription factor AP-1 [44]. The concomitant activation of NF-*κ*B and AP-1 induces a pleiotropic inflammatory response through the production of proinflammatory cytokines [44]. In addition, MyD88-dependent signalling downstream of TLR7 and TLR9 elicits the induction of IFN-*α* [44]. This response is specific to plasmacytoid dendritic cells (pDC), which express high levels of TLR7 and TLR9 and produce high levels of IFN-*α*. Upon ligand stimulation, the TIR domains of TLR7 and TLR9 recruit a complex consisting of MyD88, IRAK-4, IRAK-1, and TRAF6 [45], which binds and activates the transcription factor IRF-7 thereby inducing expression of type I IFN (IFN-*α*   and IFN-*β*)  [46]. 

In summary, the small adaptor MyD88 is at the crossroad of several signalling routes triggered by noxious agents through TLR/IL-1R receptors. For this reason, it is envisioned as a potential target to downregulate excessive immune responses. At the same time, its involvement in so many physiological responses is a challenge for the development of anti-inflammatory therapeutic agents devoid of potentially dangerous side-effects.

## 6. MyD88-Independent Signalling

Several observations have indicated the presence of MyD88-independent TLR/IL-1R signalling pathways. Although MyD88-deficient cells do not express several inflammatory cytokines upon LPS stimulation, activation of NF-*κ*B and JNK is only delayed [25]. Furthermore, induction of type I IFNs is not impaired [47]. The best characterized MyD88-independent pathway is that triggered by TLR3, which requires only TRIF as adaptor [48]. On the other hand, recruitment of TRAM is required to bridge TRIF to TLR4 (Figure 2). Thus, TLR4 is capable of activating both MyD88-dependent and TRIF-dependent signalling pathways, in a sequential process that involves the endocytosis of the TLR4 complex [49]. In particular, TLR4 first induces MAL/TIRAP-MyD88 signalling at the plasma membrane. Then, following its endocytosis into early endosomes, TLR4 activates TRAM-TRIF signalling. Once recruited to the receptor, TRIF interacts with TRAF3 to activate the noncanonical IKKs TBK1 and IKK*ε* resulting in activation of IRF3 and transcription of IFN*β*   and IFN-inducible genes [50] (Figure 2).

The more limited spectrum of action of these additional adaptors suggests that specific inhibitors of their function might exert a more selective anti-inflammatory response. On the other hand, the efficacy of such compounds might also be more limited than MyD88 inhibitors. Thus, it is at the moment unclear which member of the TLR/IL-1R superfamily is the most suitable target for pharmaceutical approaches.

## 7. Inhibition of TLR /IL1-R Function As aTherapeutic Approach

The central role of the members of the TLR/IL-1R superfamily in the immune response is highlighted by their implication in inflammatory and immune disorders such as sepsis syndrome, asthma, atherosclerosis, Alzheimer's disease, rheumatoid arthritis (RA) [21, 51]. Moreover, on a susceptible genetic background, TLR signalling can also induce autoimmune diseases such as Systemic Lupus Erythematosus (SLE), Multiple Sclerosis (MS), and Inflammatory Bowel Diseases (IBD) [52]. For these reasons, therapeutic targeting of TLR/IL-1R signalling is gaining more and more attention as a potentially valuable approach for many diseases of the immune system. Herein, we illustrate some novel strategies utilized for the development of anti-inflammatory therapeutics based on interference with the function of the TIR domain of members of the TLR/IL-1R superfamily.

One approach to modulate the activity of TLRs is the inhibition of intracellular proteins involved in the signalling pathways triggered by multiple receptors [53]. This view has been criticized on the ground that global inhibition of TLR signalling might be deleterious, as it could lead to a reduction in the body's defences against pathogens [54]. However, recent evidence suggests that the redundancy of mammalian host's immune responses together with the high degree of cross-talk between TLR-initiated signalling pathways might allow to overcome a generalized block in the immune response [55]. For instance, children with recurrent pyogenic infections display inactivating mutations in either the DD or TIR domain of MyD88 that render the patients vulnerable to *S. pneumoniae*, *S. aureus*, and *P. aeruginosa*. However, these patients are normally resistant to most common bacteria, viruses, fungi, and parasites [56]. Thus, although MyD88 is involved in almost all TLR signalling pathways, suppression of its function does not cause a complete block in the immune response. Similar findings were reported with IRAK-4-deficient patients [57]. Albeit these deficiencies are life-threatening in the childhood—with about 40% mortality in the first eight years of life—they progressively become less severe with age. Indeed, no deaths or invasive infections were observed in patients over the age of 8 and 14 years, respectively [58]. The improved clinical status was not due to any leakiness in MyD88 and IRAK-4 deficiency, suggesting that the MyD88-dependent TLR/IL-1R signalling plays a vital role early in life, but becomes less important for survival during ageing. This is likely consequent to activation and/or maturation of TLR-independent innate immunity [9, 59–62]. Moreover, these findings seem to suggest that innate immunity is more important upon the very first encounter with a pathogen. Once adaptive immunity is generated, however, resistance to infection becomes quite efficient even in the absence of crucial functional components of TLR signalling [63].

**Figure 4:**
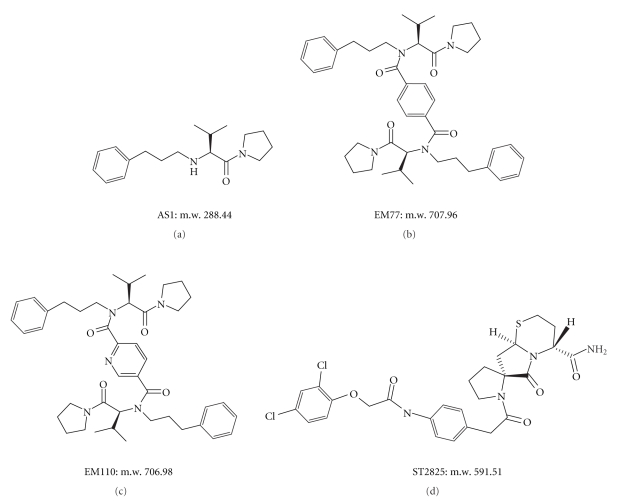
Chemical Structure of TIR BB-Loop Mimetic Compounds. AS1 (a), EM77 (b), and EM110 (c) inhibit the association between IL-1R and MyD88 [64, 65]. ST2825 (d) inhibits MyD88 homodimerization and its interaction with IRAK1 and IRAK4 [66].

TLR-mediated signalling is of paramount importance in eradicating microbial infections and promoting tissue repair. Nevertheless, it must be tightly regulated [67] in order to prevent a sustained, overzealous activation that might set the ground for autoimmune and inflammatory disorders [68, 69]. Therefore, therapeutic agents targeting the TLR signalling must be able to antagonize the harmful effects resulting from TLR hyperactivation while sparing their properly operating functions in host-defence. In spite of these apparent obstacles, evidence is accumulating that drugs targeting TLRs and their signalling adaptors can provide new therapeutic opportunities to prevent or treat human inflammatory and autoimmune diseases [70–72]. Herein, we will focus on approaches aimed at developing rationally-designed inhibitors (Figure 4) that interfere with protein-protein interactions of adaptor-adaptor or adaptor-TLR complexes. The readers are referred to several recent reviews that discuss additional approaches that are currently under development to target TLR function [73–76].

## 8. Targeting the TIR BB-Loop for Development of Novel TLR/IL1-R Signalling Inhibitors

Protein-protein interactions are central to most biological processes, suggesting that interfering with specific interactions might affect cellular responses. Nevertheless, developing small molecules that modulate these interactions may not be an easy task, due to typical flatness of the interface of contact between proteins and because large surface areas are usually involved [77, 78]. However, despite this approach presents a major challenge in terms of therapeutic feasibility, initial steps have been taken with the design of peptide-based inhibitors. 

The TIR domain of TLR/IL-1R proteins is a putatively suitable target. In particular, the BB-loop region may be regarded as a critical functional interface of TIR domain for its critical role in proper signalling [10, 15, 79].

## 9. BB-Loop Decoy Peptides

Decoy peptides are short amino acid sequences of a protein that are expected to mimic its interaction surface and to prevent interaction of the prototype proteins with their partners. Several reports have shown the successful realization of this concept, and a number of decoy peptides binding to BB-loops were found to inhibit TLR/IL-1R signalling. 

A TIRAP decoy peptide consisting of the 14 amino acid-long sequence in the BB-loop (LQLRDAAPGGAIVS), fused to the *Drosophila* antennapedia homeodomain to facilitate the intracellular delivery [80], specifically blocked TLR4-induced activation of NF-*κ*B without affecting the TIRAP-independent TLR9 response [27]. In vivo administration of TIRAP inhibitory peptide counteracted the lung inflammatory response in healthy C57BL/6 mice [81]. The peptide abolished LPS-induced TNF-alpha, IL-6, and IL-8 expression in alveolar macrophages, whereas it attenuated *E. coli*-induced expression of these cytokines and chemokines [81]. These results have suggested new therapeutic options for TIRAP inhibitors in the treatment of acute lung injury and acute respiratory distress syndrome. 

Similarly, BB-loop heptapeptides derived from MyD88 and IL-18R inhibited homomeric interaction of MyD88 TIR domain or full-length MyD88 in vitro [82]. These peptides exerted a specific effect, because heptapeptides derived from BB-loop of other TLR/IL-1R proteins were either less effective (TLR1) or completely inactive (IL-1RAcP). Moreover, a cell permeable derivative of the MyD88 BB-loop decoy heptapeptide (RDVLPGT) significantly reduced IL-1-induced NF-*κ*B reporter activity and blocked MyD88 homomeric interaction in live cells [82]. A number of studies have confirmed the activity of this construct in different experimental settings. For instance, this MyD88 inhibitory peptide significantly suppressed HMGB1-induced IL-23 release in alveolar macrophages by significantly inhibiting IRAK4 activation [83]. It was also found that this decoy peptide diminished MyD88-dependent MMP-13 gene expression, phosphorylation of MAPKs, and AP-1 activity in normal human knee articular chondrocytes [84], suggesting a possible application of this approach to treatment of RA. Moreover, the MyD88 inhibitor peptide specifically reduced TNF-*α* production and Poly(g-Glutamic acid) nanoparticles (NPs)-induced DC maturation [85]. Other authors reported that preincubation of professional antigen-presenting cells (APCs) with this molecule almost completely inhibited induction of CD80 expression by either human *β*-defensin-3, an antimicrobial peptide, or LPS. Remarkably, the MyD88 inhibitory peptide had minimal and nonsignificant effects on costimulatory molecule induction by IFN-*α*, indicating its specific action in TLR-induced APC differentiation [86].

By following the same experimental approach, Toshchakov and colleagues performed systematic investigations of decoy cell permeable peptides containing TIR domain BB-loop sequences derived from the adaptor proteins MyD88, TIRAP, TRAM, and TRIF as well as the receptors TLR1, 2, 4, and 6 [87–89]. These decoy peptides were all able to inhibit, with varying activity, the TLR signalling pathways [88]. Although the TLR2 and TLR4 decoy peptides also showed some degree of cross-reactivity, they did not interfere with TLR3 signalling [89]. Notably, BB-loops of TLR4 and TLR3 share only five identical amino acids, with proline 712 present in TLR4 but not conserved in TLR3, hence providing a possible structural base for the lack of effects of TLR2- and TLR4- derived decoy peptides toward TLR3 signaling.

Thus, the studies reported above highlight the possibility to produce inhibitory drugs that interfere with protein-protein interactions in the TLR/IL-1R signalling pathways.

## 10. BB-Loop Peptidomimetics

The BB-loop decoy peptides may also represent a valuable starting point to produce synthetic small molecules that mimic the structure of target proteins, hence paving the way for developing novel therapeutic agents.

Bartfai and colleagues were the first to show that the BB-loop region of the TIR domain was amenable to development of selective synthetic inhibitors of protein-protein interactions. By focusing on TIR-domain interactions between IL-1RI and MyD88, they synthesized a low-molecular-weight molecule mimetic, hydrocinnamoyl-L-valyl pyrrolidine. This molecule is based upon the protruding three amino acid residues of the MyD88 BB-loop, which mimic the (Phe/Tyr)-(Val/Leu/Ile)-(Pro/Gly) sequence [64], consensus for several TLR/IL-1R family members [90]. This mimetic compound blocked IL-1*β*-induced phosphorylation of the mitogen-activated protein kinase p38 in EL4 thymoma cells [64]. Moreover, sandwich ELISA assays demonstrated that this compound inhibits the IL-1*β* mediated association of IL-1R1 and MyD88 in both EL4 cells and in freshly isolated lymphocytes from mouse spleen. The disruption of the IL-1RI-MyD88 interaction was also shown to be selective over other TLR members. Remarkably, the inhibitory effects on IL-1*β*-signaling were confirmed in vivo, as mice treated with 200 mg/kg of the compound exhibited significant attenuation of the IL-1*β*-induced fever response [64]. The same TIR BB-loop mimetic was investigated in vivo in a myocardial ischaemia model, and it was shown to decrease infarct size by ~33% and to improve ejection fraction and fractional shortening in treated mice [91]. These results suggested that modulation of the IL-1R/MyD88 interaction could be a strategy for reducing myocardial ischaemic injury, and additional recent investigations support this notion [92, 93].

Based on this compound, Bartfai and colleagues synthesized a novel series of bifunctional BB-loop mimetics. Their rationale stemmed from the assumption that bifunctional compounds might be more effective blockers of protein-protein interactions than monofunctional compounds [94]. They reported that two such mimetic compounds, EM77 and EM110, possessed antinflammatory and neuroprotective properties. They inhibited MyD88-dependent proinflammatory action of IL-1*β* without affecting activation of the kinase AKT/PKB, which depended on PI3-kinase activation through binding to IL-1R [65]. The selectivity of action of the MyD88 BB-loop mimetics toward the two pathways activated by IL-1*β* in primary cultures of preoptic area (POA)/AH neurons allowed the authors to suggest that they may exert antinflammatory effects while concomitantly promoting neuronal survival in the nervous system.

The MyD88 BB-loop heptapeptide [82] also served as a template for the design and synthesis of a peptidomimetic library [95]. The RDVLPGT (Arg-Asp-Val-Leu-Pro-Gly-Thr) region was subdivided into three distinct portions: a charged portion (Arg-Asp amino acids), a hydrophobic portion (Val-Leu amino acids), and a *β*-turn portion (Leu-Pro-Gly-Thr amino acids). A peptidomimetic library consisting of 4368 direct and 234 retroinverse mimetics was designed by combining all these building blocks. For practical reasons, a subset of 83 compounds selected from the library was prepared on solid phase by using a polymer supported (aminomethyl) polystyrene (Rink amide) resin [95]. All selected compounds for synthesis met the “rule of five” [96]. The ability of the peptidomimetics to inhibit protein-protein interaction was first assessed by a yeast 2-hybrid assay [95]. Active compounds were then further validated in a mammalian cell system by evaluating the inhibition of MyD88-dependent NF-*κ*B activation. One of the most effective compounds, termed ST2825, inhibited homomeric interaction of MyD88 TIR domains [66]. This effect was specific for TIR domains and did not affect interaction of MyD88 DDs. Moreover, ST2825 blocked recruitment of IRAK1 and IRAK4 by MyD88, leading to inhibition of IL-1*β*-mediated NF-*κ*B activation. ST2825 also blocked TLR9-elicited signalling pathways by suppressing B cell proliferation and differentiation into plasma cells in response to CpG. Additionally, oral administration of ST2825 in mice dose-dependently inhibited IL-1*β*-induced production of IL-6 [66]. Finally, ST2825 intraperitoneal administration significantly protected against left ventricular (LV) enlargement in a permanent ligation model of acute myocardial infarction in mice [97].

These findings [91, 97] suggest that MyD88 inhibition may represent a completely novel approach for future translational investigations for the prevention of heart failure following acute myocardial infarction [98]. Nevertheless, a generalised suppression of MyD88 function might cause unwanted side effects, especially in chronic diseases that require continuous treatments. Thus, a controlled suppression may prove to be a viable therapeutic approach in an anti-inflammatory therapy once an inflammatory condition is presented. This notion is supported by studies conducted using RDP58, a novel anti-inflammatory d-amino acid decapeptide that inhibits the MyD88 pathway by disrupting the formation of the MyD88/IRAK4/TRAF6 complex [99]. Indeed, early human trials have shown an improvement in mild-to-moderate ulcerative colitis [100], and RDP58 is currently being developed in clinical trials for IBD (http://www.genzyme.com/corp/licensing/genz_p_rdp58_login.asp).

## 11. Conclusions

A tremendous progress has been made over the past several years in elucidating signalling pathways involved in inflammatory disorders, pointing to NF-*κ*B as the crucial downstream player. An immediate and transient activation of NF-*κ*B is important for the normal physiological response to pathogenic damage, but its persistent and excessive activation is conducive to development and progression of cancer and chronic inflammatory disorders [101, 102]. Although much emphasis has been placed on the development of NF-*κ*B inhibitors [103], generic inhibition of NF-*κ*B may lead to undesired side effects. Hence, a challenging objective is to develop drugs that block its effects in specific pathways, while leaving its physiological functions in other contexts largely intact. TLR/IL-1R pathways seem to respond to these requirements. They represent attractive targets for anti-inflammatory drug discovery, because their inhibition may impair a subset of noxious inflammatory signals impinging on NF-*κ*B, while sparing its normal physiological activation [104]. Blockade of adaptor proteins connected to these signalling pathways, such as MyD88, is expected to be more effective than inhibition of individual ligand activities, due to the mechanistic sharing of a common transduction pathway [53, 55]. In particular, the blockade of TIR-TIR interactions between various members of the TLR/IL-1R superfamily provides new opportunities in light of the highly conserved nature of the TIR domain.

Although several reports have shed light on the structures of TIR domains from human TLR/IL-1R proteins [10, 14, 105], Protein Data Bank (PDB) IDs: 2JS7; 2Z5V, their homomeric and heteromeric interactions have not been fully elucidated yet. Recent investigations suggest that following ligand-induced interaction of TIR-containing receptors a multi-TIR complex may form upon recruitment of multiple cytoplasmic adaptors [37, 106]. A major goal is the development of specific antagonists able to dismantle assembly of these signalling platforms. Despite such an approach presents daunting challenges in terms of therapeutic feasibility, initial steps have been taken by designing inhibitor decoy peptides that block the function of adaptors [81, 82]. However, it has to be underlined that these peptides will unlikely form per se the basis for new drugs, but chemists may use them as templates to develop peptidomimetics or other compounds. The recent identification [64, 66, 94] of a few TIR mimetics (Figure 4) allows to envision that design of further selective inhibitors of TIR-domain-containing proteins may be within reach. Yet, determining how to maintain the balance between host-defence functions and the undesired effects that may result from TLR inhibition remains a serious issue for those designing new therapeutics. Additional clinical experience with these novel molecules might allow to establish their relative safety and efficacy in human beings. Hopefully, these novel therapeutics may not only find application in acute settings, such as septic shock, but also in the treatment of autoimmune disorders characterised by recurrent episodes of inflammatory flares.

## Abbreviations

AP-1: activator protein 1
IL-1R: interleukin (IL)-1 receptor
IL-18R: IL-18 receptor
IL-1RAcP: IL-1 receptor accessory protein
IL-18RAcP: IL-18 receptor accessory protein
IRAK: IL-1-receptor-associated kinase
IRF: interferon regulatory factor
JNK: c-Jun N-terminal kinase
MyD88: myeloid differentiation primary response protein 88
MD-2: myeloid differentiation factor-2 (synonym of LY96, i.e., lymphocyte antigen 96)
IKK: inhibitor of NF-*κ*B kinase
NF-*κ*B: nuclear factor *κ*B
NEMO: NF-*κ*B essential modulator
TAB: TAK1 binding protein
TAK1: transforming growth factor *β*-activated kinase-1
TBK1: TANK (TRAF family member-associated NF-*κ*B activator)-binding kinase 1
TIRAP: (TIR domain-containing adaptor protein)
TLRs: Toll-like receptors
TRAF: TNF-receptor-associated factor
TRIF: Toll/IL-1-receptor-domain-containing adaptor inducing interferon-*β*
TRAM: TRIF-related adaptor molecule
